# QuantPrime – a flexible tool for reliable high-throughput primer design for quantitative PCR

**DOI:** 10.1186/1471-2105-9-465

**Published:** 2008-11-01

**Authors:** Samuel Arvidsson, Miroslaw Kwasniewski, Diego Mauricio Riaño-Pachón, Bernd Mueller-Roeber

**Affiliations:** 1 Max-Planck Institute of Molecular Plant Physiology, Am Mühlenberg 1, 14476 Potsdam-Golm, Germany; 2 Institute of Biochemistry and Biology, University of Potsdam, Karl-Liebknecht-Straße 24-25, Haus 20, 14476 Potsdam-Golm, Germany; 3 Department of Genetics, University of Silesia, Jagiellonska 28, 40032, Katowice, Poland

## Abstract

**Background:**

Medium- to large-scale expression profiling using quantitative polymerase chain reaction (qPCR) assays are becoming increasingly important in genomics research. A major bottleneck in experiment preparation is the design of specific primer pairs, where researchers have to make several informed choices, often outside their area of expertise. Using currently available primer design tools, several interactive decisions have to be made, resulting in lengthy design processes with varying qualities of the assays.

**Results:**

Here we present QuantPrime, an intuitive and user-friendly, fully automated tool for primer pair design in small- to large-scale qPCR analyses. QuantPrime can be used online through the internet  or on a local computer after download; it offers design and specificity checking with highly customizable parameters and is ready to use with many publicly available transcriptomes of important higher eukaryotic model organisms and plant crops (currently 295 species in total), while benefiting from exon-intron border and alternative splice variant information in available genome annotations. Experimental results with the model plant *Arabidopsis thaliana*, the crop *Hordeum vulgare *and the model green alga *Chlamydomonas reinhardtii *show success rates of designed primer pairs exceeding 96%.

**Conclusion:**

QuantPrime constitutes a flexible, fully automated web application for reliable primer design for use in larger qPCR experiments, as proven by experimental data. The flexible framework is also open for simple use in other quantification applications, such as hydrolyzation probe design for qPCR and oligonucleotide probe design for quantitative *in situ *hybridization. Future suggestions made by users can be easily implemented, thus allowing QuantPrime to be developed into a broad-range platform for the design of RNA expression assays.

## Background

The use of real-time quantitative PCR (qPCR) [1] in medium – (hundreds of transcripts) to large-scale (thousands of transcripts) profiling experiments is growing. While in a large number of experiments qPCR is still mainly used to confirm results obtained by microarray-based hybridization experiments, the number of high-throughput discovery experiments is growing steadily [2,3], especially for the quantification of transcripts of low abundance (e.g. those coding for transcription factors), due to the low detection limit of the method [4].

There are surprisingly few free software packages available to the academic research community suitable for the design of primer pairs for such high-throughput projects, for online use or download, including Osprey [5], Primique [6] and a few interfaces to Primer3 [7] such as Primer3Plus [8], AutoPrime [9], BatchPrimer3 [10]. Additionally, some databases of pre-computed primers, RTPrimerDB [11], PrimerBank [12], qPrimerDepot [13], AtRTPrimer [14] and DATFAP [15], have been established. There are numerous commercial and free software packages available for low-throughput design of primers, some of which are highly configurable and well suited for the design of primer pairs for qPCR.

However, none of the available packages combines all the important features (strict parameters for primer design, strict specificity checking and targeted design to avoid problems with contaminating genomic DNA) into a simple pipeline. Instead, with currently available computational tools, users have to either manually move information (such as identifiers, transcript sequences, primer sequences and others) between software packages or perform some steps completely on their own, such as specificity checking using an alignment package like BLAST [16]. Such manual steps make researchers loose valuable time, increase the risk of mistakes (e.g. labeling and sequence errors), and force them to take important decisions based on their personal interpretation of complex problems regarding large amounts of data (such as BLAST alignment sets), which either require expert knowledge or introduce bias into the results. With respect to the available primer pair databases, they are usually of limited scope. Often, only few species are covered (human and mouse being clearly over-represented), few transcripts of the species are represented (especially in databases based on submitted or published primer pairs), or inappropriate primer design parameters for combined analysis were used, requiring time-consuming optimization of PCR amplification conditions.

Here we developed QuantPrime, a program for design and specificity testing of primer pairs for qPCR, designed to meet the needs of the average or advanced user in low- to high-throughput transcript profiling experiments, while keeping the user interface very simple and yet providing important features missing in other available software packages and web services.

## Implementation

QuantPrime includes a relational database for information storage, scripts containing the procedures to perform primer pair design and specificity testing, scripts for sequence installation and maintenance, scripts for command line user interface used in high-throughput design, and a web interface as the main user interface for low- to medium-throughput primer design. For academic users we currently offer web access to the public QuantPrime server (available at ) or, on demand, compiled scripts for local installation. Commercial users are requested to get in contact with the authors to develop a license agreement.

The public QuantPrime server is currently set up with publicly available transcriptome and genome annotations from 295 different eukaryotic species. Table 1 gives examples of supported species with included features and references. The list can be easily extended according to user requests.

**Table 1:** Examples of transcriptome annotations available on the public QuantPrime server

	**Annotated features included in QuantPrime**				
**Species**	**Genomic sequences**	**Splice variants**	**Keyword search**	**Annotation source**	**Reference**
254 different species or crosses	No	No	Yes	TIGR plant transcript assemblies	[22]
91 different species or crosses	No	No	Yes	UniGene	[23]
*Arabidopsis thaliana*	Yes	Yes	Yes	TAIR release 7	[24]
*Aspergillus niger*	Yes	No	No*	JGI assembly v1.0	Non-published data
*Bos taurus*	Yes	No	Yes	NCBI RefSeq	[25]
*Chlamydomonas reinhardtii*	Yes	No	No*	JGI assembly v3.1	[26]
*Danio rerio*	Yes	No	Yes	NCBI RefSeq	[25]
*Drosophila melanogaster*	Yes	Yes	Yes	FlyBase release 5.4	[27]
*Homo sapiens*	Yes	No	Yes	NCBI RefSeq	[25]
*Homo sapiens*	Yes	Yes	Yes	H-Invitational Database 5.0	[28]
*Mus musculus*	Yes	No	Yes	NCBI RefSeq	[25]
*Oryza sativa ssp japonica*	Yes	Yes	Yes	TIGR release 5	[29]
*Ostreococcus lucimarinus*	Yes	No	No*	JGI assembly v2.0	Non-published data
*Physcomitrella patens ssp patens*	Yes	No	No*	JGI assembly v1.1	[30]
*Populus trichocarpa*	Yes	No	No*	JGI assembly v1.1	[31]
*Rattus norvegicus*	Yes	No	Yes	NCBI RefSeq	[25]
*Saccharomyces cerevisiae*	Yes	No	Yes	Saccharomyces Genome Database	[32]
*Selaginella moellendorffii*	Yes	No	No*	JGI assembly v1.0	Non-published data
*Vitis vinifera*	Yes	No	No	Genoscope assembly	[33]
*Xenopus tropicalis*	Yes	No	Yes	NCBI RefSeq	[25]

The latest versions of the annotations were added, and are updated regularly as updates become available.

* Protein IDs are searchable.

### User interface

The web interface is designed for maximum simplicity and convenience for the user. Users have to register at the first time they visit the website. The registration step allows users to return at a later time to check the results of longer runs. Their gene lists and jobs are kept confidential, i.e. no information is relayed to other users. Furthermore, registration eases the even distribution of computing resources among users and it is the main mechanism to verify academic affiliation. An account with access to limited computing resources is available for testing purposes.

The work flow starts with the generation of a 'Project' that is associated with the annotation of a species and a certain quantification protocol. The quantification protocol implies certain parameters for primer design and specificity testing; four standard protocols for typical situations are provided:

1. SYBR Green-based real-time qPCR (accept splice variant hits): typical parameters for real-time qPCR are used, such as 50–150 bp amplicon length, 60°C annealing temperature and strict primer criteria for G/C content and melting temperature (Tm). The specificity testing will allow amplicons present in splice variants of the transcript (more details in the 'Work flow' section).

2. SYBR Green real-time qPCR (no splice variant hits): as 1, but no amplicons in splice variants of the transcript are allowed.

3. End-point semi-quantitative PCR (accept splice variant hits): similar to 1, except that longer amplicons are preferred (350–1500 bp) for easier in-gel quantification.

4. End-point semi-quantitative PCR (no splice variant hits): as 3, but no amplicons in splice variants of the transcript are allowed.

Users are allowed to change any parameter and create custom protocols; see Additional file 1 for a list of all possible parameters.

Next, users should create a list of transcript identifiers in the project for which primer pair design is planned. This list can either be entered manually (using the identifiers of the chosen annotation), or can be created from a similarity-based search using BLAST and a starting query sequence. Additionally, for certain annotations, keywords describing the gene(s) can be used in a text search for identifiers.

**Figure 1:**
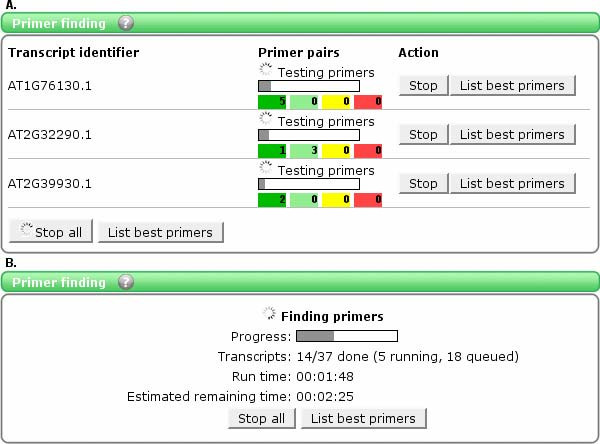
**'Primer finding' in QuantPrime**. The figure shows an example of the QuantPrime user interface for primer finding (A: up to nine transcripts, B: ten or more transcripts). The progress and success of the finding can be followed closely for small number of transcripts, for larger batches the time estimation helps users to estimate when the primer pairs will be ready.

Once the list of identifiers is ready, users may proceed to 'Primer finding' (Figure 1), which when started will continue completely in the background; in the meantime users can continue to look at resulting primer pairs or add new transcripts to the list. Larger primer finding projects may take longer time to process, therefore users may close the web browser and return at a later time to check the status of their jobs.

**Figure 2:**
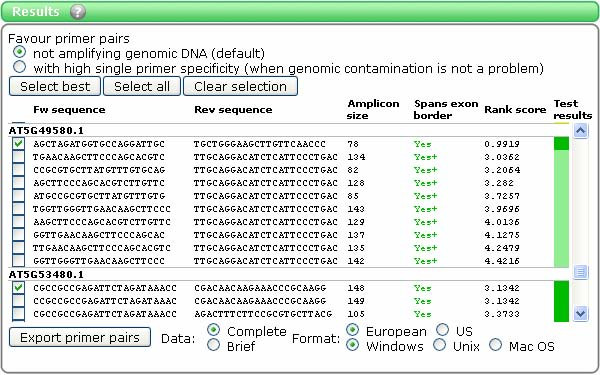
**'Results' in QuantPrime**. The figure shows an example of the 'Results' page. Primer pairs successfully identified for the examined transcripts are presented. The following information is provided: the sequences (5' to 3') of the forward and reverse primers; the amplicon size (in bp); whether at least one primer spans an exon-exon junction ('Yes' in all cases in the example shown); the rank score (as calculated by Primer3); and the color code of the specificity rank given to the primer pair (see text for details). When clicking the primer pairs, more details are shown (see Figure 3).

**Figure 3:**
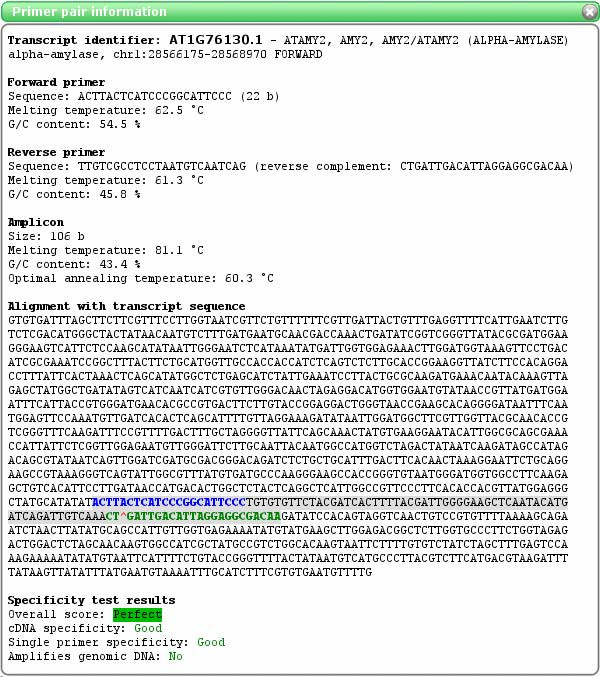
**Primer pair details in QuantPrime**. The figure shows an example of the 'Primer pair information' page. The page provides details about the selected primers and the amplicon. Positions to which the primers anneal within the target sequence are indicated in blue or green; the amplicon is highlighted by gray shadowing. Primers shown in blue anneal to an exon, whereas primers shown in green anneal across an exon-exon junction (the position of the intron is indicated by a red arrow head). In the 'Specificity test results' section, details about the specificity of the primer pair can be seen. If specificity problems exist, more details can be found here concerning the other possible amplicons.

Successful primer pairs are displayed in the 'Results' page (Figure 2), where users can inspect primer pairs in detail (Tm, G/C content, positions within transcript sequence etc., see example in Figure 3) and do bulk export of the primer data (in delimited plain text format) for ordering or local storage.

Users may return at a later time to access their data, as lists of transcripts and primer pairs are automatically saved into their corresponding projects. On the public server, projects are kept for at least a month after the latest update, and may then be deleted by the administrator for space limitation reasons. Thus, users are recommended to export primer data and store locally for reference purposes.

### Work flow

QuantPrime employs a fully automated work flow for design and specificity testing of primer pairs, a process that does not require any intermediate intervention by the user. Once users have added the transcript identifiers to the project, selecting the 'Start' button will initiate the whole primer selection process, and the identified primer pairs will automatically be displayed in the 'Results' page when the process is completed.

**Figure 4:**
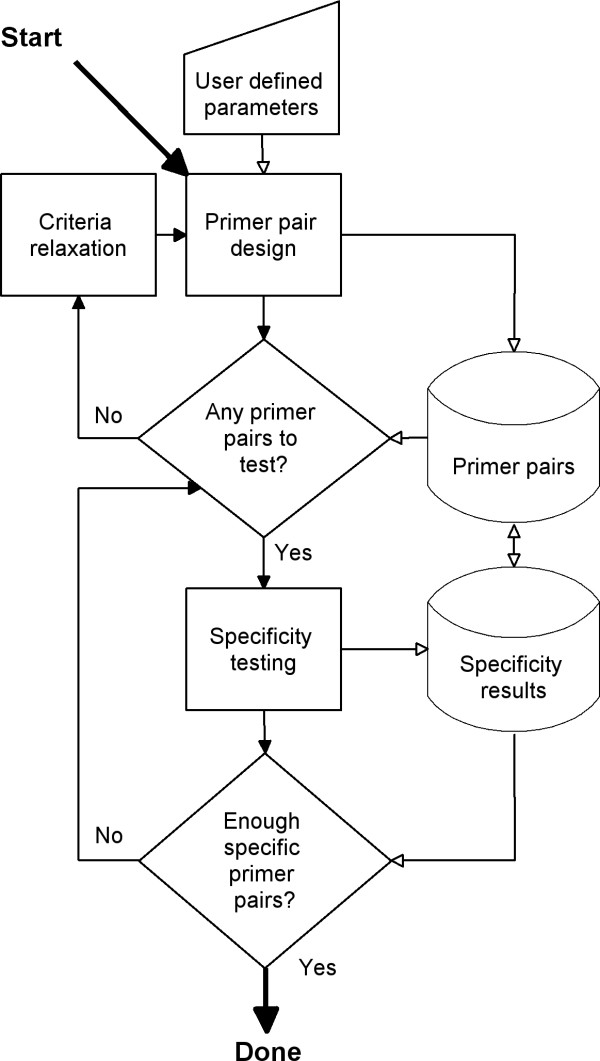
**Overall work flow of primer pair design and specificity testing**. Filled arrows symbolize logical flow while open arrows symbolize data flow.

The overall work flow of QuantPrime is sketched in Figure 4. It has two main algorithms, one for primer pair design and one for specificity testing, which are accessed by worker threads which check the output of each algorithm and decide upon the measures to be taken. The worker threads operate independent of the web server, processing submitted jobs according to defined load balancing principles (distributing computing power equally between users and projects). Due to the loosely bound system architecture it is straightforward to attach additional computing nodes to the central database allowing for high user loads. For testing purposes, a developer machine was set up to work as a computing node for the public server. With rising demand on the public server, local computing resources can be quickly mobilized to avoid long waiting times for the end users.

**Figure 5:**
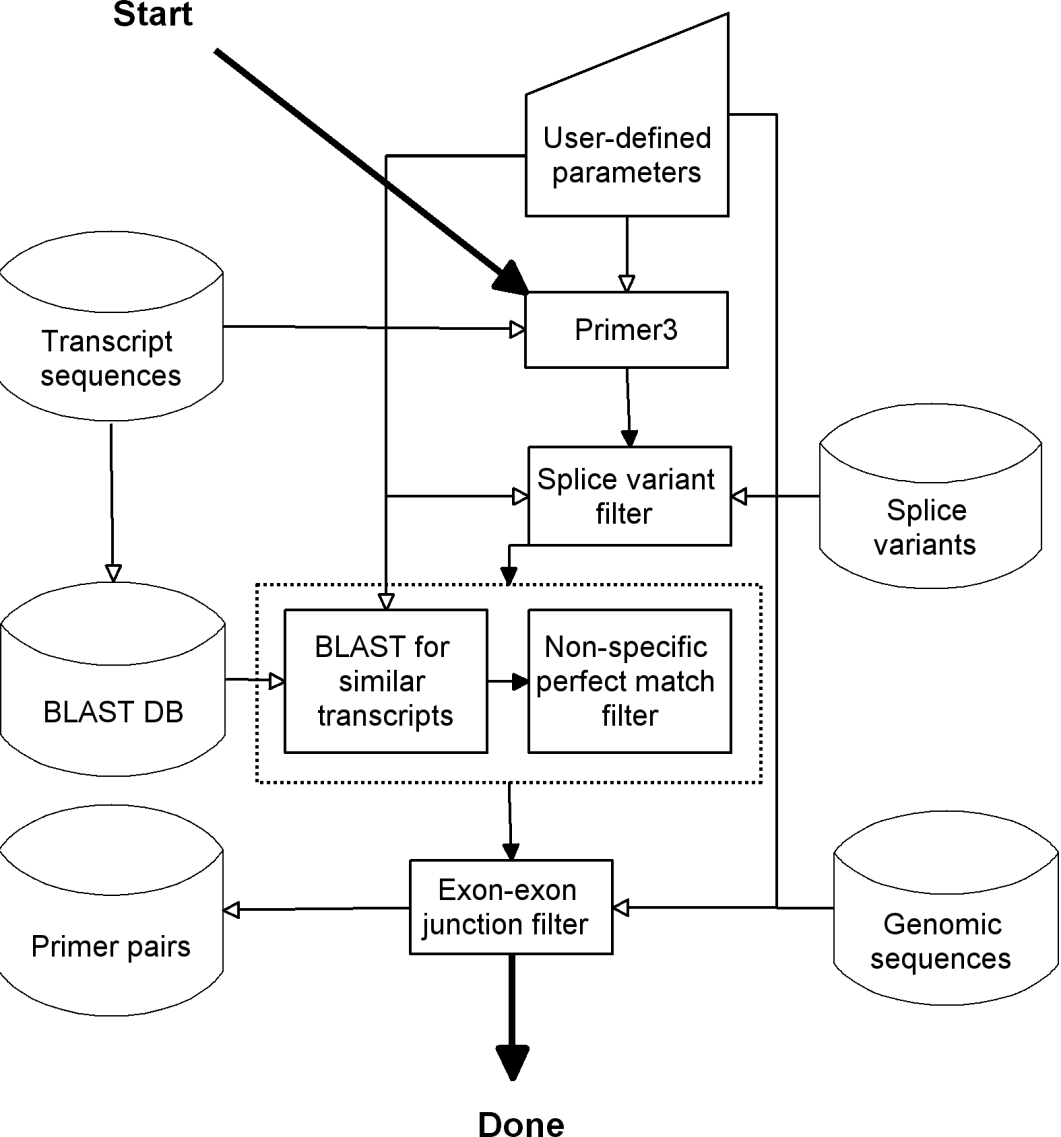
Work flow overview of the primer pair design algorithm.

The primer pair design algorithm uses the Primer3 software to design primer pair candidates; a graphical representation can be found in Figure 5.

The Primer3 design parameters can be specified by the user when setting up the project; default settings are as follows:

● Primer length: 20–24 bases

● Amplicon size: 60–150 bp

● Primer melting temperatures (Tm): 64 +/- 3°C (for optimal annealing around 60°C) (using nearest neighbor thermodynamics [17]), maximum 2°C Tm difference between forward and reverse primers

● Amplicon melting temperature: 75–95°C

● G/C content: 45–55%

● Max. repetition of a nucleotide: 3

● G/C-clamp: last 3' base of each primer must be a G or a C

In addition to the Primer3 selection criteria, the primer pair candidates are filtered through the following steps:

● Extended G/C clamp options: to avoid mispriming, it is often appropriate to avoid too many G/C bases within the 3' region of the primer. This cannot be controlled by Primer3; therefore we introduced a parameter that allows the user to define a maximum number of G/C bases to be present in the last 3' bases. The default setting is maximum three G/C bases in the last five bases of a primer.

● Amplicon bias at 3' end of transcript: primers for amplicons at the 3' end of the transcript (the last 1000 bp) are favored. For the common user this is often wanted as cDNA preparations primed with oligo-d(T)_x _generally exhibit 3' region bias. For those using random hexamers for cDNA synthesis, this parameter can be switched off.

● Skip 3' UTR: in cases where multiple polyadenylation signals exist in the 3' UTR it might be desirable to avoid priming in this region, as it could lead to biased quantification. This option can be switched on for custom design protocols.

● Exon-exon junction in primers: as RNA preparations may contain some genomic DNA even after digestion with DNAse I, such primers can successfully distinguish between cDNA and genomic DNA. When possible (i.e., when a genomic sequence with one or more intron(s) is available), primers that span an exon-exon junction are favored, especially when the junction occurs at the 3' end of the primer, to further decrease the probability of extendable annealing to genomic DNA.

● Specificity pre-filtering: in order to save workload for the specificity testing algorithm, obvious unspecific primer pairs are removed at this step. This is achieved by finding transcripts that are similar to the target transcript using BLAST (blastn of transcript against the whole transcriptome with an e-value = 1) and filtering out the primer pair candidates annealing perfectly to any of those sequences. Three configurations of the filter are possible; one that forces the algorithm to find primer pairs amplifying all splice variants of the transcript (for annotations containing such information), one that forces it to find only those specific to a certain splice variant, and one that allows (but does not force) them to amplify other splice variants (default setting).

**Figure 6:**
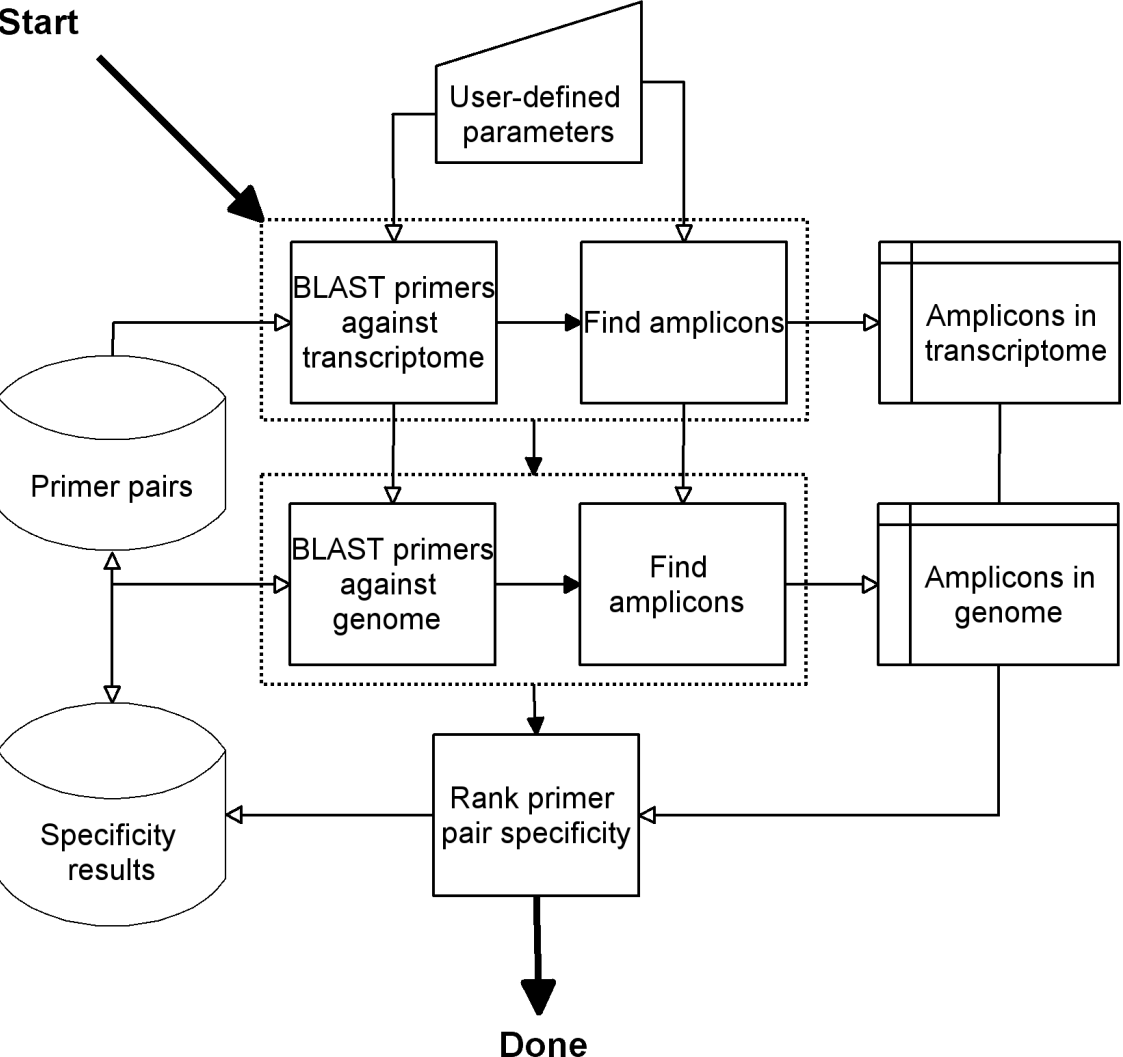
Work flow overview of the primer pair specificity testing algorithm.

The successful primer pairs are saved to the database, and the algorithm reports the number of designed primer pairs back to the calling worker thread. If it was possible to find primer pairs, the next step is specificity testing, described below (an overview is shown in Figure 6):

The primer pair specificity determination algorithm is based on the interpretation of BLAST results (with default settings: e-value = 200, word size of 7), using each primer as a query towards the transcriptome and, when available, against the genome. To identify unspecific amplicons in a transcriptome or a genome, the following (configurable) criteria are applied to the BLAST hits:

● Last two bases of the 3' region of each primer must be identical to the BLAST hit.

● Amplicons of up to 1500 bp are considered for SYBR Green protocols, and 3500 bp for end-point protocols.

Even though the primer pairs cannot give rise to an unspecific amplicon, it is generally preferred that they should be as specific as possible to the target sequence. This is approximated by checking whether a single primer in the pair has a significant (the default setting is 75%) identity to another cDNA sequence, and where the last 3' base is identical (which can be configured).

The information from the above procedures is assembled and saved into the primer pair database. Based on this specificity information, QuantPrime labels the tested primer pairs with one out of four specificity ranks: bad, acceptable, good or very good. They are defined as follows:

1. Bad (shown in red in the web interface): might amplify a non-specific cDNA fragment.

2. Acceptable (yellow): amplifies only the specific sequence, but one primer has a high similarity with a non-target sequence **and **the primer pair might amplify genomic DNA.

3. Good (light green): amplifies only the target sequence, but one primer has a high similarity with a non-target sequence **or **the pair might amplify genomic DNA. This is the highest possible rank for primer pairs designed for species without a genome annotation.

4. Very good (dark green): amplifies only the target sequence, both primers are highly specific to this sequence and will not amplify genomic DNA.

The list of designed primers is worked through until enough (the default setting is 10) of at least acceptable (rank 2) primer pairs are found. The worker thread then decides whether it is possible to find higher-ranking primer pairs (e.g., when more primer pairs spanning exon-exon junctions can be designed); if so it continues until it is successful or until a certain primer pair threshold is reached (default setting is 500 primer pairs).

The work flow implemented on the web server only performs automated relaxation in amplicon 3' bias and exon-exon junction criteria; the Primer3 parameters are not relaxed. Thus, for certain transcripts, QuantPrime will fail to find specific primer pairs; with the default settings, we arrived at a failure rate of 2–9% (see Table 2). If the user wishes to relax the Primer3 parameters to be able to find specific primers for such problematic transcripts, a new project has to be created with different primer design parameters. Some users might find this procedure cumbersome, but we chose this design to prevent primer pairs with heterogeneous design parameters to be mixed within an assay. We are open for user suggestions to introduce certain configurable relaxations in future versions of QuantPrime.

**Table 2:** Results of *in silico *benchmarking of QuantPrime

				**Primer pair specificity ranking^1^**		
**Species**	**Transcripts**	**Total search time**	**Average search time**	**Acceptable^2^**	**Good^3^**	**Very good^4^**
*Arabidopsis thaliana*	5000	20:22:06	15 s	4916 (98%)	4323 (86%)	2534 (50%)
*Vitis vinifera*	5000	50:45:33	37 s	4765 (95%)	3927 (78%)	2315 (46%)
*Drosophila melanogaster*	5000	13:48:45	9.9 s	4894 (97%)	4075 (81%)	3096 (61%)
*Chlamydomonas reinhardtii*	5000	12:11:07	8.8 s	4568 (91%)	3999 (79%)	2349 (46%)
*Oryza sativa ssp japonica*	5000	83:31:12	60 s	4658 (93%)	3821 (76%)	1984 (39%)
*Hordeum vulgare*	23078	22:56:59	3.6 s	22145 (95%)	21564 (93%)	-

Primer pairs designed for hypothetical high-throughput experiments, for random transcripts of each species. The numbers of successfully designed primer pairs for the different specificity ranks and the search times are reported for each species (percentages refer to the total number of transcripts tested).

^1 ^Percentages indicate for how many of the transcripts primer pairs of at least the rank given were identified. ^2 ^'Acceptable' amplifies only the specific sequence, but one primer has a high similarity with a non-target sequence and the primer pair might amplify genomic DNA. ^3 ^'Good' amplifies only the target sequence, but one primer has a high similarity with a non-target sequence or the pair might amplify genomic DNA. This is the highest possible rank for primer pairs designed for species without a genome annotation. ^4 ^'Very good' amplifies only the target sequence, both primers are highly specific to this sequence and will not amplify genomic DNA.

## Results

### Experimental testing of primers designed through QuantPrime

To verify the experimental usefulness of the primer pairs designed with QuantPrime, we tested it to design primers for a medium-sized expression profiling experiment for *Arabidopsis thaliana *(for 128 transcripts of various genes), carried through by fellow researchers in our group. The default settings for design and specificity testing (SYBR Green protocol, splice-variant hits allowed) were used and the highest ranking primer pairs were chosen. As can be seen in Table 3, we experienced a success rate of 96%, meaning unique amplicons of predicted size and amplification efficiencies (E) = 1.8 (see Methods for details). Over 88% of the primers were predicted not to amplify genomic DNA. For five out of 128 transcripts we obtained non-satisfying results. For those, good primer pairs could be obtained by testing one or two alternative primer pairs designed by QuantPrime, without having to perform any PCR optimization (results not shown).

**Table 3:** Experimental results of primer pairs designed with QuantPrime

**Experiment**	**Predicted gDNA-safe**	**Quality control passed^1^**	**Quality control passed^1 ^for detectable transcripts^2^**
*A. thaliana*	113/128 (88.3%)	117/128 (91.4%)	117/122 (95.9%)
*C. reinhardtii*	24/33 (72.7%)	28/33 (84.8%)	28/29 (96.6%)
*H. vulgare*^3^	-	27/30 (90.0%)	27/28 (96.4%)

	**137/161 (85.1%)**	**172/191 (90.1%)**	**172/179 (96.1%)**

The primer pairs were designed for wet-lab quantification experiments. The number of primer pairs passing strict quality control checks (melting curve analysis, agarose gel separation and efficiency check) are reported in the table.

^1 ^Melting curve analysis, gel analysis and efficiency check (E ≥ 1.8) passed. ^2 ^Undetectable transcripts (Ct > 40) removed from the statistics. ^3 ^TIGR Transcript Assembly annotation used, no genomic sequences available.

We also designed primer pairs for 33 transcripts (cell cycle genes) from *Chlamydomonas reinhardtii *and tested them in the same way as above. In this case transcripts of four genes could not be detected, and as the primer pairs for these transcripts spanned exon-exon junctions, we could not test them on genomic DNA. However, only one of the primer pairs of the detectable transcripts did not pass the quality control (having multiple products seen on gel), giving a success rate of 97%. Seventy-three percent of the designed primer pairs were predicted not to amplify genomic DNA.

Additionally, primer pairs for 30 different barley (*Hordeum vulgare*) transcripts were tested. For two primer pairs, no product could be detected, but only one of the detectable transcripts did not pass the quality control (low amplification efficiency), yielding a success rate of 96%. As no whole-genome sequence is available for barley, no predictions for genomic amplicons could be made.

In these three experiments, we thus observed a success rate > 96%. Examples of primer pairs and PCR amplification products separated on agarose gels can be found in Additional file 2.

To assess QuantPrime's accuracy of prediction of genomic DNA amplification, 173 primer pairs from an existing qPCR platform for tonoplast-related transcripts of *A. thaliana *(to be published elsewhere)were tested *in silico *with QuantPrime and experimentally with genomic DNA in real-time PCR. QuantPrime predicted 95 of these as 'gDNA-unsafe', while in real-time PCR measurable amplification was recorded for 88 of the primer pairs (data not shown). Twelve primer pairs (6.9%) were falsely predicted as 'gDNA-unsafe', and 19 (11%) falsely as 'gDNA-safe'.

### *In silico *benchmarking of QuantPrime

In order to assess the success rate and speed of QuantPrime for larger expression profiling projects, hypothetical high-throughput assays were designed for six different species. Five assays consisted of respectively 5000 randomly selected transcripts from current genome annotations of five species (*Arabidopsis thaliana, Vitis vinifera, Drosophila melanogaster, Chlamydomonas reinhardtii *and *Oryza sativa *ssp.*japonica*), while the sixth assay consisted of the whole UniGene collection of barley (*Hordeum vulgare*) transcripts. As seen in Table 2, the success rates (primer pairs ranked as 'acceptable' or better by specificity testing) varied between 91 and 98%, which correlates relatively well with the status and complexity of the annotations. For the higher specificity ranks rather high variation between species was observed, ranging from 76–93% for the rank 'good', and 39–61% for the rank 'very good'. Since the barley annotation lacks genomic information, 'good' is the highest possible rank. Primer pair identification speed varied between 3.6 (barley) and 60 (rice) seconds per transcript, correlating roughly with the size of the sequence sets to be searched by BLAST.

We also did preliminary tests with data sets from larger transcriptomes/genomes (human, mouse), for which the design speed dropped (data not shown). This is due to a higher memory demand of the BLAST runs that can be offered in the future, when requests for the service rise.

## Discussion

Our experimental results show that the primer pairs designed by QuantPrime can be directly used with a high success rate (> 96%) in qPCR applications, without a need for experimental optimization of individual reaction conditions. When running tests in parallel on a standard desktop computer, the speed is enough to design primers for high-throughput projects for small- to medium sized transcriptomes as shown by the *in silico *tests.

To our knowledge, there are no other web-based tools directly comparable to QuantPrime, although programs like Osprey [5] and Primique [6] offer possibilities for batch primer pair design. In those two other applications, however, the user has to supply the database against which primer pair specificity is tested, but the upload capacity is limited to 10 MB which does not suffice for most transcriptomes. QuantPrime always tests the primer pairs against the whole transcriptome of the annotation used, and additionally offers a richer user interface, exon-exon junction design of primers to avoid genomic DNA amplification, and a high degree of customization of parameters, features not available in the other software packages. Most annotations are already included in QuantPrime; in the case that users have special annotations not available on the public server, they can contact us for adding it there, or they can run QuantPrime locally. A more exhaustive comparison of QuantPrime with other available primer design software can be found in the Additional file 3.

For some species pre-computed databases of primers exist. An example is AtRTPrimer [14] containing primer pairs for most genes of *A. thaliana*. When looking at the available primers in this resource one will find that the parameters for design, especially amplicon size, make the primer pairs unsuitable for real-time PCR, and due to the differences in Tm between different primer pairs exhaustive PCR optimization would be necessary for using them in high-throughput. The authors report a success rate of 93%, however only 21 primer pairs offered by the database were experimentally validated. In comparison, QuantPrime offers complete customization of parameters for different quantification methods, and we see higher success rates (> 96% for the three species tested here, n = 191). Another example is the PrimerBank [12], which covers primer pairs for human and mouse transcripts, which could be useful for high-throughput purposes (due to strict design criteria), even though amplicon sizes vary. Those two databases are limited to specific species; there are a couple of databases covering more species, notably RTPrimerDB [11], which however cover very few non-human genes. Another database containing primer pairs for plant transcription factors is DATFAP [15], which however is based on EST sets, which is questionable for *A. thaliana *and *O. sativa *for which good genome annotations are available. It therefore lacks information about possible genomic sequences amplified by the primer pairs; additionally Tm values vary widely between primer pairs, which might require exhaustive PCR optimization.

The parameter flexibility for design and specificity testing offered in QuantPrime makes it straightforward to employ it for the design of oligonucleotides for a number of other quantification applications, such as qPCR with hydrolyzation probes (e.g. TaqMan probes, Scorpion primers), quantitative *in situ *hybridization of mRNA and others. Such protocols will be added to QuantPrime as we gather experimental data and feedback from users.

## Conclusion

The QuantPrime website offers a unique service to the scientific community, with ease-of-use, flexibility of parameters and a broad scope of transcript databases and genomic annotations, which should make it a very useful tool for primer design. No other publicly available tool offers the same services. Overall, the speed of computation and the quality of the designed primer pairs as shown experimentally make QuantPrime (on the public web server or as standalone software) a suitable system for primer design in low- to high-throughput transcription profiling projects.

We are open for suggestions from the scientific community to further develop QuantPrime in the future. Upon request we may for example include further transcript databases and genome annotations, sets of parameters for other quantification protocols and applications, or improve the applied specificity testing algorithms. Institutions wanting to host mirrors of the QuantPrime public web server or supply additional computing power are encouraged to contact the authors.

## Methods

### General

Standard molecular techniques were performed as described [18]. Oligonucleotides were obtained from MWG (Ebersberg, Germany). Unless otherwise indicated, other chemicals were purchased from Roche (Mannheim, Germany), Merck (Darmstadt, Germany), or Sigma (Deisenhofen, Germany).

### Growth conditions

*Arabidopsis thaliana *(L.) Heynh accession Col-0 plants were grown in growth chambers with an 8-h day length provided by fluorescent light at 120 μmol m^-2 ^s^-1 ^(50% intensity during the first and last 30 minutes of the light period) and a day/night temperature of 20/16°C and relative humidity of 60/75%. Whole, young plants (four weeks after germination) including washed roots were harvested 2 hours after lights-on, snap-frozen in liquid nitrogen and stored at -70°C until RNA extraction. *Chlamydomonas reinhardtii *CC503 cw92 mt+ was grown under continuous light (100 μmol m^-2 ^s^-1^) at 21°C in HEPES-based medium as described [19]. *Hordeum vulgare *(Karat variety) plants were grown as previously described [20], and parts of roots from seven days-old seedlings were used for total RNA extraction.

### RNA extraction and cDNA synthesis

After grinding of plant/algal material in liquid nitrogen, total RNA was isolated with Trizol reagent (Invitrogen, Karlsruhe, Germany) or RNeasy Plant Mini Kit (Qiagen, Hilden, Germany) following the manufacturers' specifications. RNA quality was determined spectrometrically (A_260_/A_280 _> 1.8) using a NanoDrop ND-1000 spectrometer (NanoDrop, Detroit, USA) and by visual inspection of separated bands on agarose gels.

After isolation, genomic DNA was digested using Turbo DNA-*free *recombinant DNAse I (Applied Biosystems Applera, Darmstadt, Germany) following the manufacturer's specifications. The level of remaining genomic DNA contamination was measured by diluting the samples to the same concentration as the final cDNA samples (10 ng μl^-1^) and performing real-time PCR using primers for a genomic sequence (*UBQ10: *Fw 5'-GGCCTTGTATAATCCCTGATGAATAAG-3', Rev 5'-AAAGAGATAACAGGAACGGAAACATAGT-3'). Samples with consistent cycle threshold (Ct) values below 35 were re-treated with DNAse I or new RNA extractions were performed.

Two μg of total RNA was used in 20-μl reactions for cDNA synthesis, using RevertAid R-minus cDNA synthesis kit (Fermentas, St. Leon-Rot, Germany), following the manufacturer's specifications. The cDNA was then diluted 1:10 in order to reduce the effect of RNA isolation and cDNA synthesis buffer on the subsequent PCRs.

### Real-time quantitative PCR

qPCR was carried out in technical triplicates or quadruplicates using 0.5 or 1 μl of diluted cDNA in 5- or 10-μl reactions, 2 or 4 μl of 500 nM primer pairs and 2.5 or 5 μl of 2× Power SYBR Green PCR Master Mix (Applied Biosystems). The following PCR protocol was used on Applied Biosystems 7300 (96-well plates) and 7900HT (384-well plates) real-time PCR systems: 10 min at 95°C, 15 sec at 95°C, and 1 min at 60°C repeated in 50 cycles, followed by melting curve analysis. When testing primer pairs, the PCR products were then separated on a 2% agarose gel and visualized with ethidium bromide, using 50 bp DNA ladder (Invitrogen) for size determination.

Cycle threshold (Ct) values for each reaction were calculated using Applied Biosystems SDS software, with baseline set to cycle 3–15 and threshold to 0.2 Rn, recorded from the SYBR Green I dye signal normalized against the ROX dye signal.

Real-time PCR amplification efficiencies were calculated using the LinRegPCR tool [21], using the best-fit method for 4 to 6 points. This tool uses linear regression on log-values of normalized fluorescence data from individual reactions to calculate E in the equation for PCR kinetics, N_C _= N_0 _* E^C^, which states that the amount of product after C cycles (N_C_) is equal to the starting concentration (N_0_) times the efficiency (E) to the power C; 100% efficiency would give an efficiency value of 2.

Efficiency values from fitted curves with R-squared values below 0.999 were considered as unreliable; Ct values and efficiencies from such reactions were removed from further calculations. Medians of Ct values and efficiencies were calculated and used in further calculations.

### Public server setup

The web-based QuantPrime program runs on a Linux-based server, with two Intel 1.6 GHz QuadCore 64-bit processors and 4 GB of RAM, configured to run up to six design/testing threads in parallel, always leaving two virtual processors available for database and web handling. This was found to be the most efficient configuration for this single server; setting up the program and database in a clustered environment with specialized data and computation nodes should lead to synergistic speed improvements, as the amount of data transferred between database and executing threads are kept very low.

### *In silico *benchmarking

For the random selection of transcripts from annotations, the built-in random function in MySQL was used to order all transcripts from the respective annotation having a transcript length of more than 300 bp, of which the top 5000 were selected.

The run times given are real time (not CPU time), meaning the difference of the time point when the experiment started and when it finished. The average time per transcript is the total time divided by the number of transcripts. Due to the parallel nature of the program, the typical time to design one specific primer pair for a transcript is longer.

## Availability and requirements

**Project name: **QuantPrime

**Project home page: **

**Operating systems: **Platform independent

**Programming languages: **Python and PHP (web interface)

**Other requirements: **Web browser (supporting JavaScript) for using the public server; for standalone use: BioPython 1.4 or higher, MySQL 5.0 or higher, Primer3 1.1.1 or higher, NCBI BLAST 2.2.13 or higher

**Any restrictions to use by non-academics: **License needed

## Authors' contributions

SA designed and programmed QuantPrime, carried out most of the primer testing and drafted the manuscript. MK designed the graphical user interface and contributed to the design of the program, carried out the tests with barley and revised the manuscript. DMRP helped out to design the program, prepared sequence databases, installed and administrates the public server and revised the manuscript. BMR supervised the group, helped out with the design and testing of the program and helped drafting the manuscript. All authors read and approved the final manuscript.

## Supplementary Material

Additional file 1 **List of customizable parameters in QuantPrime**. A comprehensive list of all parameters that can be customized in QuantPrime, with parameter ranges and default values. Click here for file

Additional file 2 **Examples of primer pairs with gel images**. Examples of primer pairs for different species with images of agarose gel separations of their PCR amplification products. Click here for file

Additional file 3 **Comparison of QuantPrime with other primer design software**. A comparison table including QuantPrime and other commonly used primer design software. Click here for file
