# Supplementary material for: QuantPrime – a flexible tool for reliable high-throughput primer design for quantitative PCR
Source: BMC Bioinformatics. 2008 Nov 1;9:465. doi: 10.1186/1471-2105-9-465 (PMC2612009; doi:10.1186/1471-2105-9-465)

Examples of primer pairs

In the figures PCR amplification products separated on agarose gels (1 x TBE, 2 % agarose, 35 minutes separation at 100 Volt) are seen. The first and last lane of each gel contain DNA size markers of 200,150,100 and 50 bp (top to bottom).

***Arabidopsis thaliana* (A and B)**

No	Transcript	Forward primer sequence 5'-3'	Reverse primer sequence 5'-3'	Amplicon size
1	AT2G38620.1	AGAGCTCCTGAAGTTTTGCTTGGT	TTGCCTCCTAATCATCTCGGCA	96
2	AT3G54180.1	TGGTTCGGAGGCAAGCTCTTTT	TCAGTTGGTGTTCCTAGCAACCTG	85
3	AT4G37490.1	ACCAGCACTCTCAAGCATCACAC	GCCAACAGCTTTGCACAGTCCAT	68
4	AT1G09960.1	AGCATCGTGTTTGCTGCTGTGT	TGGGACGCTGTAAGTTATCGCC	72
5	AT1G19450.1	GGAGTTGGCGTTGTTCAGGTAGT	AAGCCGACGACCTGCTTTATCC	72
6	AT1G53210.1	CCATCATCTTCGCATCCCGCAA	TGTCACTCCACCACATAGCTCG	74
7	AT2G40610.1	AGCCAGCTTTTCTTCAGATCGCT	TGGTACTCTTCGGAAAGAGACAGG	68
8	AT3G03220.1	TGCCGGTGCAGTATCGAAGGAT	TCGACTGTAAACCGCATGCTCC	61
9	AT3G15370.1	TGATAGCCCTGCTTCACTTGGAGG	AATCCGGCGTGGTACGGATTGT	60
10	AT3G29030.1	ACTTTTGTCCTCCTGGTGGTGC	CACTTCTCTTGCACCGAACCCTT	138
11	AT3G55500.1	TGCCCGTTTTCCTCAAGATCGC	ACTCTTCCTACATGCCACCCTG	80
12	AT4G38210.1	GATAGCCACAGCCACGCTTTCT	ATACCCACAAGCGCCTCCAGTA	73

***Chlamydomonas reinhardtii* (C)**

No	Transcript	Forward primer sequence 5'-3'	Reverse primer sequence 5'-3'	Amplicon size
1	BIS_CDKI	AGAGGTTAGCCAGGTGTCTCTAGC	GTTGCGAATGTGGATGCGCTTG	79
2	BIS_DP	GTTTCTGCCCTTCATCCTGGTG	TCTGGAACGGCGAGTAGAAGTC	116
3	Chlre2_kg.scaffold_21000008	AAATCGTGCGCTCGGAAATCCAC	CATGAGACCCGTCAAGTCGAAGTC	95
4	Chlre2_kg.scaffold_4000140	AACCACCTCCCGGCTTGTTAAG	TCGTACACCCAGTCGGGTAACTTG	63
5	Chlre2_kg.scaffold_50000017	CCCAAGCAGATGATGGAGTACCTG	AACTCGCTGCTGAACACCTTCTC	71
6	Chlre2_kg.scaffold_71000018	TCAAATCCGCGCTCAACGTACC	GCGTCCAGCTGAGACTTATCATCG	64
7	estExt_fgenesh2_kg.C_160082	GTGCAACCAACAACGCACGAAG	GTTGCTCGACCAATCCAATGCC	80
8	estExt_fgenesh2_kg.C_560016	ATGTACTACCTGCGCACCAAGG	GCTCATGGAGGGCATCTTCATCTG	141
9	estExt_fgenesh2_kg.C_90002	CGCCTTCTATCAGAGAACGAGTGG	CCGCCTGAACAGCATGATATGTGG	108
10	estExt_fgenesh2_pg.C_10108	TGCCCTACGTCATCATCAAGGC	CAGGTAGTGCTGCACGTCAATG	110

Examples of primer pairs

***Hordeum vulgare* (D)**

No	Transcript	Forward primer sequence 5'-3'	Reverse primer sequence 5'-3'	Amplicon size
1	Bmic7	GCATTTGTGCATGTGTGTCATCTC	TGGCCTTATTACAAGCAGCAGGAG	65
2	Bmic8	TTTGTGTACGCGCGCCTGATAG	GTCAGTCGGATGCACAAAGGAC	66
3	Bmic9	CATCAGATTGAGCATCCAGTCCAG	TCGTCGCCTCCATTATGCTCAC	67
4	Bmic10	TCAGCTACGCCAGCTATGAAGC	AGCACCCTCTTGATCAGGTCACTG	76
5	Bmic13	AGTGCAAGCACTTCCTCTCTGAC	GCGAAATGGTTCGACCACCAAC	65
6	Bmic14	TGCAGTAAGCGGAGGAAGATACG	ACAGGAAGCACCATTTGTCCTC	65
7	Bmic15	ACCGGTTCATGGTCTCTTGATGG	AGGAAGACCGGAAGGCACATTG	72
8	Bmic16	AACCAGTTGTCAGGGCCGATTC	TGCACAAACAGTTCCTGAAGCC	68
9	Bmic19	ATCCAAGTGCTCCACCGTCAAC	ATGCGCATGCTGTTTCGCAGAC	76
10	Bmic21	CCCGTACATACAAGTGCAAGCC	GATGCGGCAAGAGAAGGACAAC	74
11	Bmic22	ACTTCCACGCCTACACCATCTC	ATGCTGGAGTAGCCGAACATGG	142
12	Bmic23	GCTTTGCAAATGCAGGAGGAAGC	ACTCCAACGCTTGGACCTTCTG	76

Examples of primer pairs

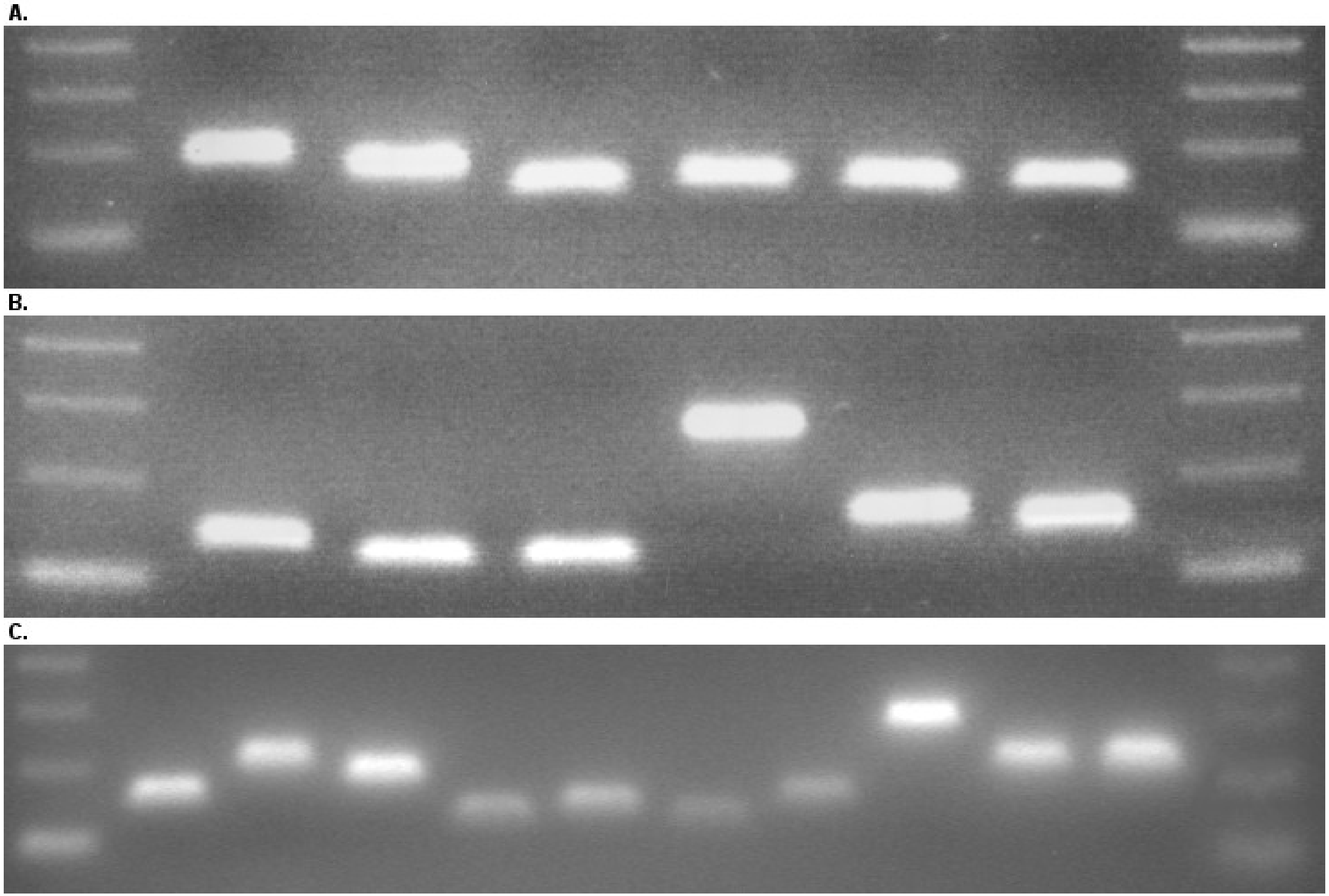

Examples of primer pairs

D.

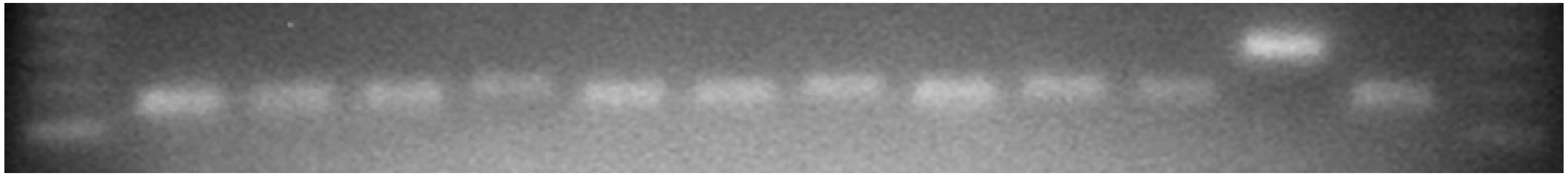

Supplement: Additional file 2 — Examples of primer pairs with gel images. Examples of primer pairs for different species with images of agarose gel separations of their PCR amplification products. [file 1471-2105-9-465-S2.pdf]
